# Prognostic significance of troponin level in 3121 patients presenting with atrial fibrillation (The NIHR Health Informatics Collaborative TROP‐AF study)

**DOI:** 10.1161/JAHA.119.013684

**Published:** 2020-03-26

**Authors:** Amit Kaura, Ahran D. Arnold, Vasileios Panoulas, Benjamin Glampson, Jim Davies, Abdulrahim Mulla, Kerrie Woods, Joe Omigie, Anoop D. Shah, Keith M. Channon, Jonathan N. Weber, Mark R. Thursz, Paul Elliott, Harry Hemingway, Bryan Williams, Folkert W. Asselbergs, Michael O'Sullivan, Graham M. Lord, Narbeh Melikian, David C. Lefroy, Darrel P. Francis, Ajay M. Shah, Rajesh Kharbanda, Divaka Perera, Riyaz S. Patel, Jamil Mayet

**Affiliations:** ^1^ NIHR Imperial Biomedical Research Centre Imperial College London and Imperial College Healthcare NHS Trust London United Kingdom; ^2^ NIHR Oxford Biomedical Research Centre University of Oxford and Oxford University Hospitals NHS Foundation Trust Oxford United Kingdom; ^3^ NIHR King's Biomedical Research Centre King's College London and King's College Hospital NHS Foundation Trust London United Kingdom; ^4^ NIHR University College London Biomedical Research Centre University College London and University College London Hospitals NHS Foundation Trust London United Kingdom; ^5^ Health Data Research UK University College London London United Kingdom; ^6^ NIHR Cambridge Biomedical Research Centre University of Cambridge and Cambridge University Hospitals NHS Foundation Trust Cambridge United Kingdom; ^7^ NIHR King's Biomedical Research Centre King's College London and Guy's and St Thomas’ NHS Foundation Trust London United Kingdom; ^8^ Institute of Epidemiology and Biostatistics University of Ulm Germany; ^9^ Faculty of Biology Medicine and Health University of Manchester United Kingdom

**Keywords:** angiography, atrial fibrillation, coronary artery disease, mortality, troponin, Atrial Fibrillation, Biomarkers, Mortality/Survival, Coronary Artery Disease

## Abstract

**Background:**

Patients presenting with atrial fibrillation (AF) often undergo a blood test to measure troponin, but interpretation of the result is impeded by uncertainty about its clinical importance. We investigated the relationship between troponin level, coronary angiography, and all‐cause mortality in real‐world patients presenting with AF.

**Methods and Results:**

We used National Institute of Health Research Health Informatics Collaborative data to identify patients admitted between 2010 and 2017 at 5 tertiary centers in the United Kingdom with a primary diagnosis of AF. Peak troponin results were scaled as multiples of the upper limit of normal. A total of 3121 patients were included in the analysis. Over a median follow‐up of 1462 (interquartile range, 929–1975) days, there were 586 deaths (18.8%). The adjusted hazard ratio for mortality associated with a positive troponin (value above upper limit of normal) was 1.20 (95% CI, 1.01–1.43; *P*<0.05). Higher troponin levels were associated with higher risk of mortality, reaching a maximum hazard ratio of 2.6 (95% CI, 1.9–3.4) at ≈250 multiples of the upper limit of normal. There was an exponential relationship between higher troponin levels and increased odds of coronary angiography. The mortality risk was 36% lower in patients undergoing coronary angiography than in those who did not (adjusted hazard ratio, 0.61; 95% CI, 0.42–0.89; *P*=0.01).

**Conclusions:**

Increased troponin was associated with increased risk of mortality in patients presenting with AF. The lower hazard ratio in patients undergoing invasive management raises the possibility that the clinical importance of troponin release in AF may be mediated by coronary artery disease, which may be responsive to revascularization.

**Clinical Perspective**

**What Is New?**

- Elevated troponin levels in patients presenting to the hospital with atrial fibrillation are associated with a high risk of mortality, with higher levels associated with worse prognosis.
- The risk of mortality associated with troponin increase was lower in patients who underwent coronary angiography than in those who did not undergo coronary angiography.

**What Are the Clinical Implications?**

- Even troponin elevations mildly above the upper limits of normal should be taken seriously in patients presenting with atrial fibrillation.
- Consideration should be given to investigate for underlying coronary artery disease in patients presenting with atrial fibrillation and an increased troponin level.

## Introduction

Patients presenting to the hospital with atrial fibrillation (AF), the most prevalent tachyarrhythmia,^1^ often undergo measurement of cardiac biomarkers.^2^, ^3^ In particular, troponin levels are measured, ostensibly, to diagnose an acute coronary syndrome (ACS) manifesting as AF.^4^ However, interpretation of the result is hampered by uncertainty over the clinical importance of troponin levels in AF. The diagnostic and prognostic utility of troponin in ACS,^5^ and other cardiac presentations, such as heart failure,^6^ is firmly established. However, these predictive relationships may not be observed in AF, where troponin release may be related to rapid ventricular response and the mechanical effects of fibrillation on the atria rather than coronary artery disease (CAD). Observational analysis of patients presenting with AF have identified associations between troponin level and clinically important outcomes, but these studies did not assess mortality. In clinical practice, small troponin increases in AF presentations are typically ignored and do not routinely prompt investigation for CAD.

The aims of our study were to explore the relationship between troponin level and mortality in patients presenting to the hospital with AF, to understand the pattern of referral for coronary angiography in relation to troponin level, and to determine the role of coronary angiography in the relationship between troponin and mortality in these patients.

## Methods

The National Institute of Health Research Health Informatics Collaborative database consists of routinely collected electronic health record data from patients attending 5 large UK tertiary care centers with emergency departments (Imperial College Healthcare, University College Hospital, Oxford University Hospital, Kings College Hospital, and Guy's and St Thomas’ Hospital) between 2010 (2008 for University College Hospital) and 2017. The data acquisition and analysis plan is found in Data S1. The National Institute of Health Research Health Informatics Collaborative study was registered at ClinicalTrials.gov, NCT03507309. This work used data provided by patients and collected by the National Health Service as part of their care and support. No verbal or written informed consent from individual patients was required for data set generation. This study was approved by the London–South East Research Ethics Committee (16/HRA/3327).

### Eligibility Criteria

We identified patients from the National Institute of Health Research Health Informatics Collaborative database who were admitted to the hospital with a primary diagnosis of AF and underwent at least one troponin measurement. Patients with a concomitant secondary diagnosis of AF were not eligible for inclusion in the study. Diagnoses were established from routinely recorded *International Classification of Diseases, Tenth Revision* (*ICD‐10*), discharge codes and were therefore established by the clinical team after inpatient investigations and management were complete. Patients meeting the eligibility criteria were followed up using routinely collected data, until death or censoring on April 1, 2017.

### Troponin Level

All analyses on troponin were performed using the peak troponin level. For patients who had a single troponin measurement, the peak troponin was based on this measurement. In the remainder of the patients who had >1 troponin test in the same hospital episode of care, the peak troponin value was defined as the highest of all measurements. For patients with multiple episodes of care for which troponin was tested, the first episode of care was used.

In clinical practice, troponin levels are frequently dichotomized into “positive” (meaning >99th percentile of the upper limit of normal [ULN] for the troponin assay) or “negative.” Furthermore, troponin levels may have a progressive relationship with prognosis, too, but the shape of this relationship is not known across the full spectrum of values; and making the assumption of a linear relationship of mortality with troponin (or log troponin) may not be secure in our study participants.

For these reasons, we treated the data in 2 ways. First, we dichotomized the results as being either positive or negative. Second, we used troponin on a continuous scale by standardizing the many troponin assays, by scaling the results using the ratio of the observed troponin value divided by the ULN for that particular troponin assay.

### Coronary Angiography and Revascularization

Patients undergoing coronary angiography, or revascularization with either percutaneous coronary intervention or coronary artery bypass grafting, during the follow‐up period were identified. To account for outpatient procedures, patients were categorized as having angiography or intervention if performed within 3 months of the peak troponin level.

### Follow‐Up

Using a retrospective cohort study design, all patients were followed up until death or censoring on April 1, 2017. Life status was ascertained using routinely collected data on the National Health Service Spine Application, which was linked to the Office of National Statistics, and thereby to the national registry of deaths.

### Statistical Analysis

Descriptive statistics are displayed as median (interquartile range) for continuous variables and number (percentage) for categorical variables. Comparisons of baseline characteristics between patients who did and did not undergo angiography were explored by Mann‐Whitney *U* test or χ^2^ test.

The relationship between dichotomous troponin level (above ULN or not), or continuous troponin, and all‐cause mortality was performed using Cox proportional hazards regression modeling, using log transformation because of the positive skew of troponin values. The proportional hazard assumption was supported by a nonsignificant relationship between the Schoenfeld residuals and time. This test was not statistically significant for each of the covariates included in the Cox regression model.

Using Martingale residuals, nonlinearity was detected in the relationship between the log hazard and all continuous covariates (age, creatinine, hemoglobin, platelet count, white blood cell count, and troponin level). To model nonlinear relationships, we used restricted cubic splines for Cox regression and logistic regression analyses to calculate mortality hazard ratio and odds of angiography outcomes, respectively. Preliminary analyses suggested that 4 unforced knots should be used to model troponin level in the restricted cubic spline analyses. Splines were adjusted for demographic characteristics, hematological and biochemical blood results, cardiovascular risk factors, and comorbidities. Subgroup analyses were performed in angiography and no angiography subgroups. Kaplan‐Meier survival curves were plotted according to angiography status.

*P*<0.05 was considered significant. Statistical analyses were performed using the R 3.5.0 statistical package (the R Core Team, Vienna, Austria). Survival analyses were performed using the Survminer and Survival R packages.

## Results

**Figure 1:**
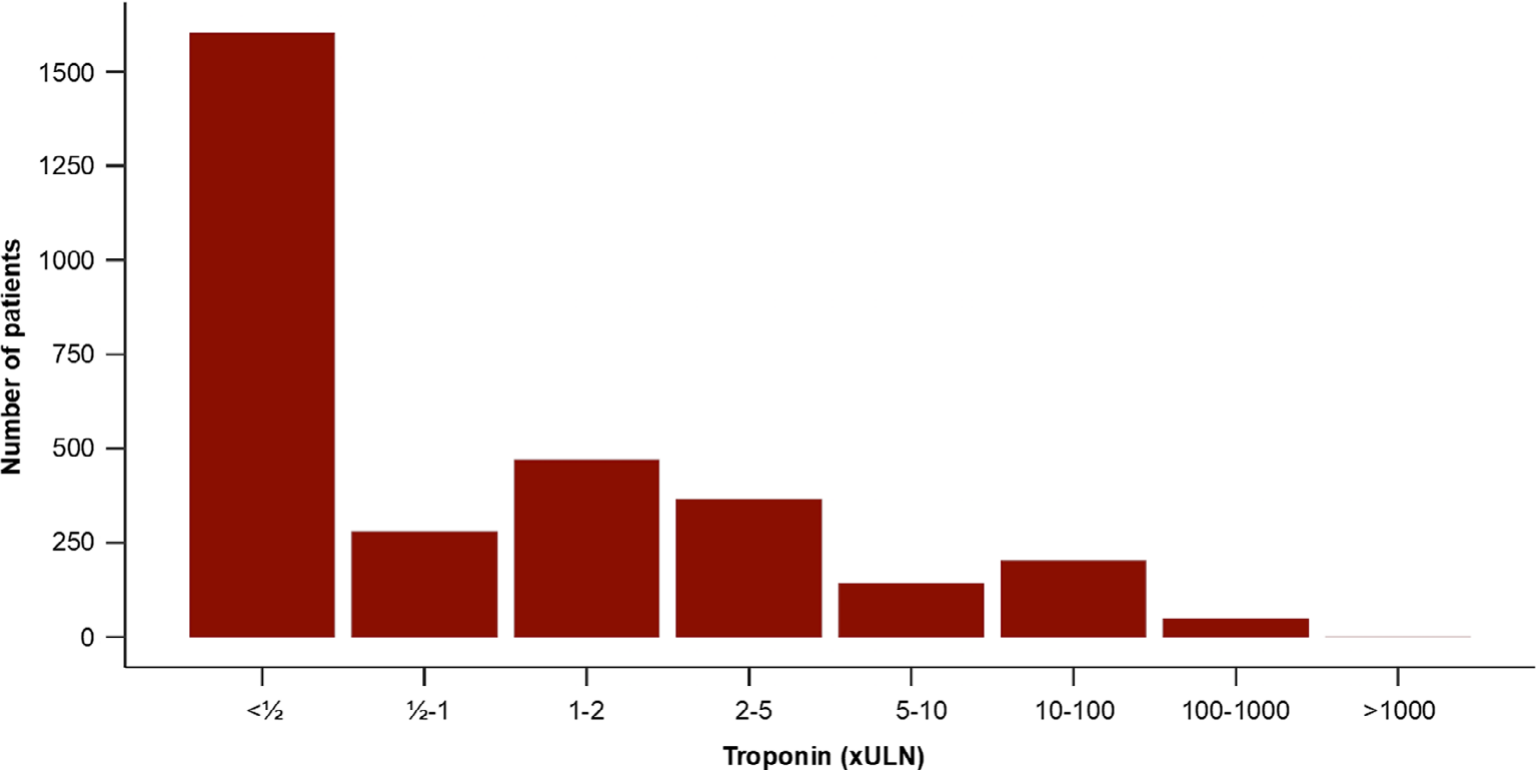
Bar chart of numbers of patients according to troponin level. xULN indicates 99th percentile of the upper limit of normal.

A total of 3121 patients admitted to the hospital with a primary diagnosis of AF, according to *ICD‐10* discharge codes, underwent troponin measurement during the study period. Their baseline characteristics are displayed in Table 1. Mean age was 73 years (95% CI, 62–82 years), and 55.7% were men. Most of these patients (60.4%) recorded a peak troponin level that was within the normal range (<1 multiple of the ULN [xULN]) (Figure 1).

**Table 1:** Baseline Characteristics of Patients

Characteristics	Patients With Primary Presentation of AF (n=3121)
Demographic characteristics	
Age, y	73 (62–82)
Men	1738 (55.7)
Hematology and biochemistry results	
CRP, mg/dL (n=2796)	5.0 (2.0–14.9)
Creatinine, μmol/L (n=3086)	82 (69–100)
Hemoglobin, g/dL (n=3075)	13.8 (12.5–15.0)
Platelet count, ×10^9^/L (n=3071)	226 (187–274)
Troponin, xULN	0.5 (0.003–2.0)
White blood cell count, ×10^9^/L (n=3075)	8.2 (6.6–10.2)
Cardiovascular risk factors	
Diabetes mellitus	355 (11.4)
Hypercholesterolemia	448 (14.4)
Hypertension	1062 (34.0)
Cardiovascular disease	
Aortic stenosis	53 (1.7)
Heart failure	302 (9.7)
Previous myocardial infarction	341 (10.9)
Other comorbidities	
Malignancy	207 (6.6)
Obstructive lung disease	146 (4.7)

Data represent median (interquartile range) or value (percentage). Numbers in parentheses indicate the number of patients who had data available for the relevant variable. AF indicates atrial fibrillation; CRP, C‐reactive protein; xULN, 99th percentile of the upper limit of normal.

### Relationship Between Troponin Level and Coronary Angiography

**Figure 2:**
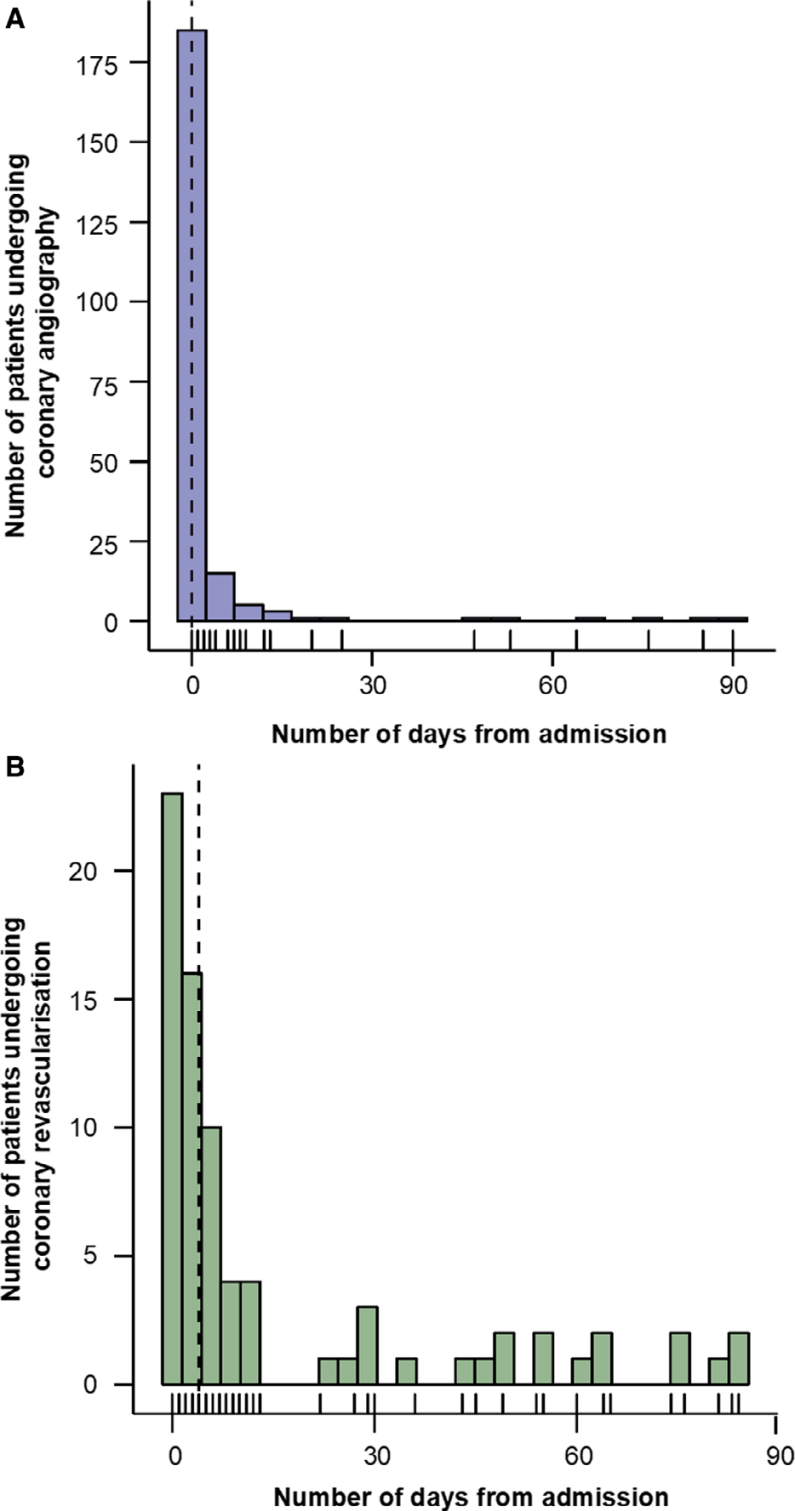
Histogram of numbers of patients undergoing coronary angiography (**A**) and revascularization (**B**) at different time points after measurement of peak troponin level at presentation.

A total of 216 patients (6.9%) underwent coronary angiography, with 78 (36.1%) of these patients subsequently undergoing coronary revascularization by percutaneous coronary intervention (93.6%), coronary artery bypass grafting (2.6%), or both (3.8%). A total of 39 patients (1.2%) had a secondary diagnosis of ACS. Most coronary angiograms (89.8%; Figure 2A) and 43.6% of revascularization procedures (Figure 2B) occurred within 72 hours of the peak troponin level. The baseline characteristics of patients who did and did not undergo angiography are displayed in Table 2. The demographic and clinical factors associated with undergoing coronary angiography are shown in Figure S1 and Table S1.

**Table 2:** Baseline Characteristics of Patients Who Did and Did Not Undergo Angiography

Characteristics	Angiography (n=216)	No Angiography (n=2905)	*P* Value*
Demographic characteristics			
Age, y	73.5 (65.3–79.0)	73.0 (63.0–83.0)	0.47
Men	144 (66.7)	1594 (54.9)	0.001
Hematology and biochemistry results			
CRP, mg/dL	6.1 (2.03–16.5)	5.0 (1.9–14.8)	0.08
Creatinine, μmol/L	84.0 (73.3–100.8)	81.0 (69.0–100.0)	0.04
Hemoglobin, g/dL	13.9 (12.5–15.0)	13.8 (12.5–15.0)	0.78
Platelet count, ×10^9^/L	224 (182–270)	226 (187–275)	0.53
Troponin, xULN	1.4 (0.003–5.6)	0.5 (0.003–2.0)	<0.0001
White blood cell count, ×10^9^/L	8.5 (6.8–10.6)	8.2 (6.6–10.2)	0.21
Cardiovascular risk factors			
Diabetes mellitus	32 (14.8)	323 (11.1)	0.12
Hypercholesterolemia	41 (19.0)	407 (14.0)	0.06
Hypertension	74 (34.3)	988 (34.0)	0.94
Cardiovascular disease			
Aortic stenosis	8 (3.7)	45 (1.5)	0.03
Heart failure	27 (12.5)	275 (9.5)	0.15
Previous myocardial infarction	65 (30.1)	276 (9.5)	<0.0001
Other comorbidities			
Malignancy	7 (3.2)	200 (6.9)	0.03
Obstructive lung disease	12 (5.6)	134 (4.6)	0.50

Data represent median (interquartile range) or value (percentage). CRP indicates C‐reactive protein; xULN, 99th percentile of the upper limit of normal.

* Comparison between angiography and no angiography groups using Mann‐Whitney *U* test for continuous variables and χ^2^ test for categorical variables.

**Figure 3:**
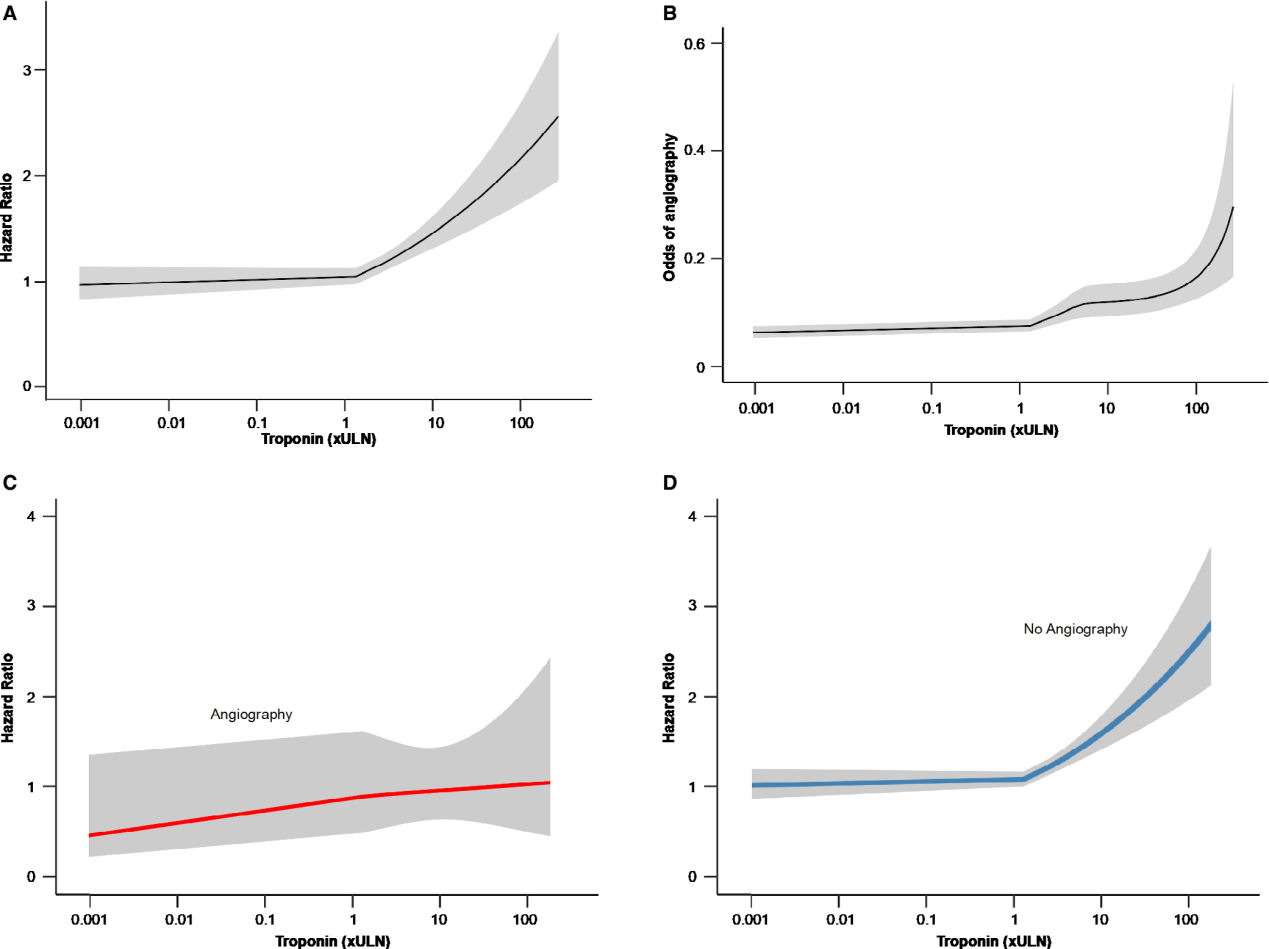
Multivariate restricted cubic spline modeling of association between troponin level and hazard ratio (**A**); association between troponin level and odds of coronary angiography (**B**); and association between troponin level and hazard ratio in angiography (**C**) and no angiography (**D**) subgroups. Data were adjusted for age, sex, CRP (C‐reactive protein), creatinine, hemoglobin, platelet count, white blood cell count, diabetes mellitus, hypercholesterolemia, hypertension, aortic stenosis, heart failure, previous myocardial infarction, malignancy, and obstructive lung disease. The shaded area denotes the 95% CI.

The relationship between troponin level and likelihood of undergoing coronary angiography was nonlinear (Figure 3B). At troponin levels less than the ULN, increasing troponin level was not associated with increasing likelihood of coronary angiography. Above the ULN, there was a direct relationship between troponin level and odds of angiography, with an exponential distribution at troponin levels >5 xULN. There was a direct relationship between odds ratio of undergoing coronary revascularization and troponin level (Figure S2). A patient with a peak troponin of 10 xULN was 2.7 times more likely to undergo coronary revascularization compared with a patient with a troponin level of 1.

### Relationship Between Troponin Level and Mortality

Over a median follow‐up of 48.1 (interquartile range, 30.5–64.9) months, there were 586 deaths (18.8%), with a 1‐year mortality rate of 4.8%. The hazard ratio for mortality associated with a positive troponin result (value above ULN) was 1.20 (95% CI, 1.01–1.43; *P*<0.05) after adjustment for key demographic and baseline clinical factors. The relationship between continuous troponin level and mortality was demonstrated using restricted cubic spline Cox regression analysis, adjusted for the same demographic and baseline clinical factors (Figure 3A). Although troponin levels <1.3 xULN showed no significant relationship with hazard ratio, at higher troponin levels, a significant positive relationship was demonstrated.

**Figure 4:**
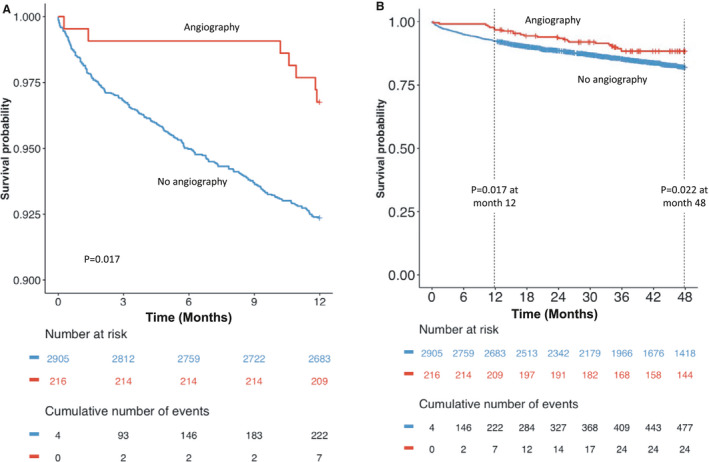
Kaplan‐Meier survival curves according to angiography status over 12 months (**A**) and 48 months (**B**) of follow‐up. Tick marks denote censored events. Survival curves compared using log‐rank statistic.

Figure 3C and 3D shows the relationship between troponin level and mortality for patients presenting with AF who underwent coronary angiography (Figure 3C) and patients who did not (Figure 3D). Although there was no significant relationship between troponin level and mortality in patients who underwent angiography (Figure 3C), a significant relationship was observed in patients who did not undergo angiography with troponin levels above the ULN (Figure 3D). Kaplan‐Meier survival analysis demonstrated worse short‐term survival in patients who did not undergo angiography (*P*=0.02; Figure 4A). This difference in mortality persisted at 4‐year follow‐up (*P*=0.02; Figure 4B). On multivariate Cox regression analysis, after adjustment for demographic and clinical factors, including troponin level, angiography was associated with a 39% reduction in mortality during follow‐up (hazard ratio, 0.61; 95% CI, 0.42–0.89; *P*=0.01). For those patients who were referred for angiography, there was a nonsignificant trend toward revascularization being associated with a reduction in mortality during follow‐up (hazard ratio, 0.36; 95% CI, 0.12–1.10; *P*=0.07). The relationship between troponin and mortality in patients who underwent angiography without revascularization and the relationship in those who did not undergo angiography are shown in Figure S3. This figure shows that increasing troponin was associated with increasing mortality risk in both groups but with wide CIs around the point estimates for unrevascularized patients who underwent angiography; it is not clear that there is a difference in the troponin‐mortality relationship between these groups.

## Discussion

This is the first study investigating the relationship between troponin level, coronary angiography, and mortality in patients admitted to the hospital with a primary diagnosis of AF. This is also the largest study to report the association between troponin level and all‐cause mortality in this group.

### Abnormal Troponin Levels Predict Mortality in AF

In 3121 patients presenting with AF, whose troponin was measured, there was a significant association between troponin levels (above the ULN) and higher mortality, even after adjusting for key demographic and clinical factors. Associations derived from dichotomizing continuous variables, such as troponin, can be driven by extreme values. However, even low‐level troponin elevations above the normal range (1–10 xULN) were associated with higher mortality risk. Abnormal troponin levels, at any level, appear to have prognostic importance in AF; and higher troponin levels appear to confer worse prognosis.

### Nonlinear Relationship Between Troponin and Mortality

The relationship between troponin level and adjusted hazard ratio for mortality in patients presenting with AF is nonlinear when considering the entire spectrum of troponin values, including detectable troponin within the normal range. Below the ULN of troponin, there does not appear to be any relationship between higher troponin levels and increased hazard ratio for mortality. A similar nonlinear pattern between troponin level and mortality has previously been observed in an unselected group of patients without ACS.^7^ Specifically, in our AF cohort, above the ULN there is a direct relationship between troponin level and mortality, reaching a hazard ratio of ≈2.6 at 263 xULN. The inflection near ULN may represent a genuine cutoff in the importance of troponin and supports the use of the 99th percentile in determining the “normal” range. Although this does not mean all patients presenting with AF should undergo invasive investigation, it does suggest that the troponin threshold for investigating for CAD may be lower than current practice.

### Coronary Angiography Is Performed at Higher Troponin Levels

Coronary angiography was performed in <7% of patients presenting with AF who had troponin measured. Below the ULN for troponin, higher troponin levels were not associated with an increased likelihood of coronary angiography. Above the ULN, there was an exponential relationship between troponin level and likelihood of coronary angiography. However, even at the highest troponin levels, less than half of patients underwent coronary angiography. This is consistent with typical clinical practice; small troponin increases in AF presentations are not deemed to be a useful marker of CAD by clinicians and even when troponin increases to high values, alternative explanations are often invoked. Other factors also influenced the likelihood of coronary angiography in this population: understandably, patients with malignancies were less likely to undergo coronary angiography and patients with prior infarcts were more likely to undergo coronary angiography. However, women were also less likely to undergo coronary angiography than men, despite other variances being accounted for in the multivariate analysis. This reflects a growing understanding that women with cardiac disease present, and are managed, in different ways to men, potentially to the detriment of women's health outcomes.

### Coronary Angiography Alters the Troponin‐Mortality Relationship

In patients who underwent coronary angiography, higher troponin levels above ULN were associated with higher mortality, but the relationship was weak, with a shallow gradient, and not statistically significant. In patients who did not undergo coronary angiography, there was a clear, direct relationship between higher troponin levels (above ULN) and higher risk of mortality. Coronary angiography appeared to be associated with a 36% reduction in mortality across the spectrum of troponin values. One possible explanation for this is that we may be selecting a relatively low‐risk group of patients for coronary angiography compared with those we choose to treat medically. An alternative explanation is that invasive management may improve the prognosis of patients presenting with AF and abnormal troponin elevations, with greater improvement at higher troponin levels. Coronary angiography at higher troponin levels is more likely to reveal clinically important CAD that is amenable to prognosis‐improving treatment. Data on the rate of medical therapy for CAD were not available in this analysis, but coronary revascularization was performed in 36.1% of patients who underwent coronary angiography and there was a trend toward improved mortality with revascularization, although this did not reach statistical significance. The utility of routinely investigating for CAD in these patients requires testing in clinical trials.

### Relationship With Existing Evidence

This is the largest study to assess the relationship between troponin and mortality in patients presenting to the hospital with AF. Other studies have assessed the effects on revascularization, stroke, cardiovascular death, and other clinically important outcomes. However, all such diagnoses, including fatal stroke and cardiovascular death, risk inaccuracy and bias to varying extents. All‐cause mortality is the only outcome where the diagnosis is free from such bias. Larger subanalyses of randomized controlled trials comparing direct oral anticoagulants with warfarin have assessed the relationship between troponin and mortality in patients with AF^3^, ^8^ and found a significant correlation. However, this is an entirely different population of patients with a *background* of AF, rather than patients presenting to the hospital with a primary diagnosis of AF. In stable patients diagnosed with AF previously, increased troponin will inevitably be predictive of poor prognosis but troponin screening for patients in a stable phase of AF is rare and not a clinically relevant problem. How to interpret troponin increases in short‐term presentations with AF is an extremely common clinical dilemma, which is addressed in this study.

Conti et al prospectively enrolled 3627 patients presenting with recent‐onset (<48‐hours) AF, but excluded patients with ACS, clinical instability, or severe comorbidity,^4^ and offered coronary angiography if the troponin level was elevated above the ULN. Troponin elevation (above the ULN) was associated with angiographic CAD, revascularization, and increased likelihood of adverse cardiovascular events, but mortality was not measured, and troponin was dichotomized at the ULN. We have shown that higher troponin levels are associated with a higher mortality risk, indicating that the magnitude of troponin increase as a continuous variable is important for decision making. Supporting our findings, Alghamry et al demonstrated, in a retrospective study of 200 patients, that troponin dichotomized at the ULN had poor ability to predict CAD, whereas peak troponin, analyzed continuously, did predict CAD.^9^ We did not find an association between higher levels of troponin below the ULN and increased mortality, despite a large number of patients analyzed at a long follow‐up duration. A smaller study (n=330) suggested detectable troponin level below the ULN does predict mortality, but this may have been a chance finding as our study population for this group of patients was 4 times larger and found no association.^10^

### Mechanisms of Troponin Release in AF

Troponin I and T bind to tropomyosin in the intracellular sarcomeric contraction complex, but they are also found in the cytosol.^11^ In ACS, obstruction of epicardial coronary arteries results in myocyte necrosis, releasing troponin into the circulation, with larger infarcts both releasing more troponin and risking worse and more likely clinical sequelae, including death.^5^ The explanation for the relationship between troponin and mortality in ACS is, thus, clear, but troponin increases carry prognostic significance in several settings, such as heart failure, pulmonary embolism, and sepsis.^6^ The cause of circulating troponin in AF is particularly disputed, however, which is one reason for the relatively low importance clinicians place on troponin leak in AF.^11^ Type II myocardial infarction caused by ventricular myocyte death during rapidly conducted AF in the context of preexisting CAD is a putative mechanism. Although it is likely that this often plays a key role, as rate control can reduce troponin leak,^12^ troponin release also occurs at normal ventricular rates. Furthermore, CAD identified is not always severe or physiologically significant, which makes treatment decisions, particularly the role of revascularization, uncertain. Although it is not known whether atrial myocytes themselves release troponin during fibrillation (regardless of ventricular rate), there is a large body of evidence for atrial scarring in AF,^12^ suggesting this may be the case. However, acute atrial necrosis may only be the mechanism for new‐onset AF as opposed to the first presentation of chronic AF. Patients with AF experience troponin increases in the short‐term phase of stroke,^13^ implicating sympathetic activation as a cause for troponin release and a marker of mortality risk. AF as a manifestation of acute type I myocardial infarction, where there is spontaneous thrombotic occlusion of coronary arteries, is thought to occur rarely. This may be more common than diagnosed and would be another explanation of the link between troponin and mortality.

We cannot directly infer, from our analysis, a mechanism for troponin release nor the mechanism via which troponin increases are associated with worse prognosis, but our findings raise the possibility that the clinical importance of troponin release in AF may mediated by CAD.

### Limitations

Although this study benefits from having been conducted using real‐world clinical data in a large number of patients from multiple centers, there are some limitations. This study was retrospective, with data extracted from electronic medical records and subject to the limitations of this approach, including difficulty in accounting for all potential confounding factors. Bias may be introduced because of inaccuracies in routine data collection. In routine clinical practice, patients with larger troponin increases are more likely to be given a primary diagnosis of ACS on discharge, even if their presentation was with AF, altering the overall risk status of the patients with AF as the primary diagnosis. The data set also includes only those patients who had a troponin measurement potentially altering the overall risk profile. Furthermore, the subtype of AF (paroxysmal or new onset) was not recorded, preventing analysis by chronicity of AF, and the role of stress testing was not available in the data set.

We could not directly infer mechanisms for the importance of troponin increases in AF. Routinely collected data from electronic health records lack resolution for fine details of clinical encounters. For this reason, we could not analyze the data for the effect of troponin on cardiovascular outcomes and compare this with all‐cause mortality. However, as previously discussed, all‐cause mortality is the only outcome to be free from bias, which is of particular relevance in observational analyses.

## Conclusions

Abnormally elevated troponin at any level is associated with increased risk of mortality in patients presenting with AF, and higher troponin levels confer worse prognosis. Coronary angiography is rarely performed unless AF is associated with large troponin increases, but when it is performed, it is associated with lower mortality. This raises the question of whether the prognostic significance of troponin could be mediated by CAD and may be responsive to revascularization. Clinical trials are warranted to clarify the role of investigating and treating CAD in patients presenting with AF with elevated troponin levels.

## Sources of Funding

This article reports independent research led and funded by the National Institute for Health Research (NIHR) Imperial Biomedical Research Centre (BRC), as part of the NIHR Health Informatics Collaborative with the NIHR Oxford BRC, the NIHR University College London Hospitals BRC, the NIHR Guy's and St Thomas’ BRC, and the NIHR Cambridge BRC. The views expressed in this publication are those of the authors and not necessarily those of the National Health Service, the National Institute for Health Research, or the Department of Health. Dr Elliott and H. Hemingway received Health Data Research funding.

## Disclosures

None.

## Supporting information


**Data S1.** Data acquisition and analysis plan.
**Table S1.** Odds Ratio of Undergoing Coronary Angiography
**Figure S1.** Odds ratio of undergoing coronary angiography.
**Figure S2.** Odds ratio of undergoing coronary revascularisation according to troponin level.
**Figure S3.** Multivariate* restricted cubic spline modelling of association between troponin level and hazard ratio for patients who underwent angiography without revascularisation (left) and those who did not undergo angiography (right).
