# Supplementary material for: Prognostic significance of troponin level in 3121 patients presenting with atrial fibrillation (The NIHR Health Informatics Collaborative TROP‐AF study)
Source: J Am Heart Assoc. 2020 Mar 26;9(7):e013684. doi: 10.1161/JAHA.119.013684 (PMC7428631; doi:10.1161/JAHA.119.013684)
Supplement: Supplementary file 1 — Data S1. Data acquisition and analysis plan. Table S1. Odds Ratio of Undergoing Coronary Angiography Figure S1. Odds ratio of undergoing coronary angiography. Figure S2. Odds ratio of undergoing coronary revascularisation according to troponin level. Figure S3. Multivariate* restricted cubic spline modelling of association between troponin level and hazard ratio for patients who underwent angiography without revascularisation (left) and those who did not undergo angiography (right). [file JAH3-9-e013684-s001.pdf]

Supplemental Material

Data S1.

NIHR Health Informatics Collaborative TROP-AF Study

DATA COLLECTION PLAN

Author: Amit Kaura
Approvers: Jamil Mayet
Date: 07/03/2017

Introduction

This document outlines the specifications and procedures for the NIHR Health Informatics Collaborative (NIHR HIC) Cardiovascular research database and defines the processes for the collection of the NIHR HIC Cardiovascular research data and onward sharing with researchers. The central research database is held within Imperial College Healthcare NHS Trust (ICHNT).

Data for all patients receiving a troponin test are collected locally at the following Trusts and submitted pseudonymously to ICHNT:

- Imperial College Healthcare NHS Trust
- University College London Hospitals NHS Foundation Trust
- Oxford University Hospitals NHS Foundation Trust
- Kings College Hospital NHS Foundation Trust
- Guys and St Thomas' Hospital NHS Foundation Trust

This document covers the processes for local collection of data at all sites. The main database and systems are hosted at ICHNT as the lead organisation for the Cardiovascular Theme.

NIHR HIC Cardiovascular Database Definitions

Local data store

Each Trust will have a local store of NIHR HIC Cardiovascular data; this collects their information in an identifiable form from clinical systems. The data will then be de-identified within the local NHS Trust. These de-identified data will be passed to the central research database.

Research database

The research database will contain only de-identified information. This database combines the data from each site (including ICHNT). The database will contain some secondary patient identifiers (e.g. date of procedure, date of death) which will not be made directly available to researchers. A further

anonymised view of the data will be prepared by ICHNT staff which will involve converting all dates to the number days since the first troponin test. This view of the data will contain only the variables necessary to complete the study rather than the full database.

Procedures for local data collection into local stores

Data will be collected automatically from primary clinical systems within each Trust. NHS staff will enter data into clinical systems during routine clinical care of patients, or data will be generated as a result of clinical tests. Data in the primary clinical systems will be processed in accordance with each Trusts clinical guidelines and subject to local quality and governance procedures. Data will be extracted automatically and validated processes for data extraction, transformation and loading (ETL) have been designed to import the data into the local secure data stores.

Access to local data stores

Access to identified data will be limited to those justified and approved by the local information governance teams and will always be NHS staff in accordance with the duty of confidentiality required by law. Anonymisation procedures will be automated after implementation.

Local data stores will be the only areas that hold any patient identifiers. De-identification is completed at this stage prior to passing data into any research databases and datasets.

De-identification

The data will be pseudonymised locally within each Trust; anonymisation processes will be automated and set up by NHS staff in accordance with:

- advice from local information governance procedures
- the HIC Standard Operating Procedure (SOP) for data sharing and anonymisation
- the Clinical Data transfer policy

The data items summarised in Table 1 will be anonymised. Anonymisation will be approved locally by information governance teams before data are sent externally to ICHNT. De-identified data are then shared in accordance with the overarching data sharing agreement.

Demographic	Anonymisation
Local Identifier	Provided if NHS number is missing
NHS Number	Use local pseudonymisation algorithm (key to be retained at source)<br>Rename field to subject ID
Family Name	To be removed – LOCAL use
Given Name	To be removed – LOCAL use
Date of Birth	YYYY
Date of Death	DD-MM-YYYY

Table 1. Anonymisation of data elements

Each site will hold two versions of the database, one identifiable and one with de-identified, pseudonymised data. The de-identified version is for use in research and shared with the central research database at ICHNT. The identifiable database is held so that if necessary patients can be re-identified if it is of importance to re-contact the patient via their care team.

NHS numbers and hospital numbers are pseudonymised using locally approved procedures. Names are removed from the dataset and date of birth is transformed to year of birth. Date of death is shared, however, is converted in to relevant survival rates on provision of data to researchers. Researchers will never see the full date of death or be able to calculate it from other information (all dates are provided as delta for first troponin). The provision of date of death has been agreed by each of the information governance offices at each of the sites in the following de-identification and anonymisation protocol for the study:

- The exact date of death is required to evaluate mortality after diagnosis.
- Patients presenting with suspected acute coronary syndromes are likely to have a high frequency of cardiac events and a high short-term mortality rate. An accurate measure of death is therefore required to fully evaluate this.
- To redact the date of death to year only would misrepresent the survival of these patients, particularly for those who survive for less than one year. The clinical leads at our BRCs have underlined the importance of having the date in full for the study.

Data validity and quality

Prior to pseudonymisation within the clinical systems, NHS number, date of birth and patient names will be automatically checked to remove duplication of patients.

Samples of data will be clinically validated by members of the clinical team to ensure that the transformation process is correct and data are attributed to the correct patients prior to pseudonymisation. After clinical validation is complete, the data will be transformed to a standardised XML format and validated (Figure 1).

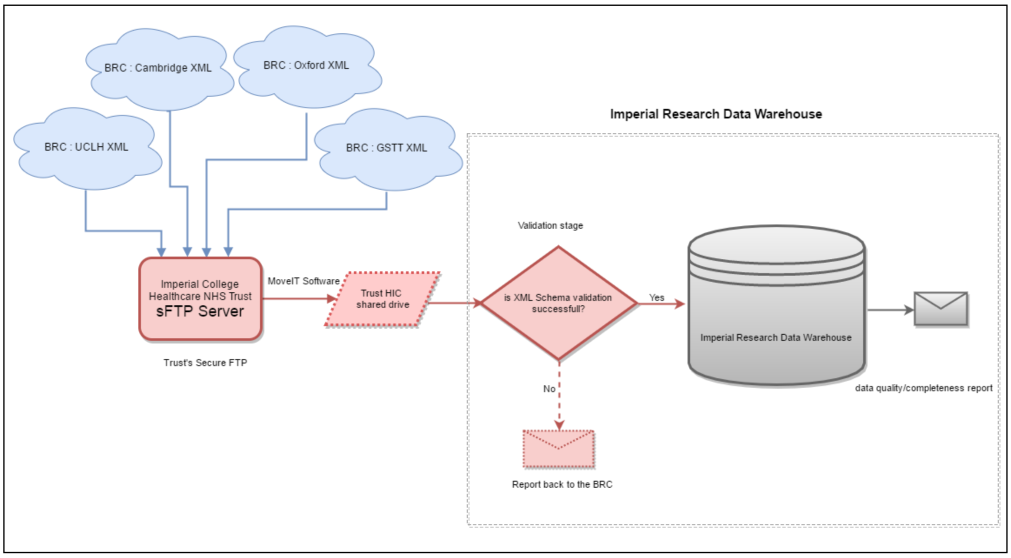

Figure 1. Data import and validation process.

Data sharing between Trusts

Data are shared in accordance with the SOP for data sharing. Data are encrypted in transit via sFTP on the N3 network (Figure 1). The sFTP is set up and hosted by ICHNT.

Procedures for secure research database

Data validity and quality

ICHNT validate the data to ensure that the structure, data items, units and data types are in accordance with the standardised data model. Data will be rejected if validation fails. On rejection, the files will be archived and the data manager will contact the data provider to review the submission and resend once corrected.

Once imported into the secure research database, data are subject to clinical validation by Cardiovascular clinical experts; these validations will be

completed by the clinical researchers using de-identified data. Data will be reviewed for data completeness, spread and actual data point values. If data appears invalid it will be rejected.

Data quality reports will be generated and provided to the research team and local data provider after each submission. These will be reviewed after each submission to ensure all areas are populated.

Research Database software

The database will be built using Microsoft SQL server 2014, Microsoft's principle database management system software. The installation of the software was carried out by certified technical consultants and tested by the Trust ICT team and data warehouse team in accordance with Trust ICT procedures and policies.

Database management

The database will be fully backed up on a daily basis. The back-ups are standardised for all Trust databases within the Trust data warehouse. Backups can be restored at any point by warehouse staff. Data are secondary copies from clinical systems; at any point the participating Trusts can re-extract the data from primary sources. Each site will submit data on a quarterly basis to the database. Data integrity checks will be completed to ensure correct structure is maintained and duplicates are not present.

Once entered on the system, data will not be changed. All access will be 'read only' except via exception, approved by the research Informatics Programme Manager and Clinical Leads group.

The database will be managed by the data manager (Ben Glampson) and developer (Abdul Mulla). Any database changes will be controlled by the research informatics Programme manager (Ben Glampson), and sanctioned by the NIHR HIC Cardiovascular scientific steering committee (chaired by Jamil Mayet). All staff are substantive NHS employees at ICHNT.

Data extracts taken for research will be stored within the data warehouse and retained for the period specified in the data request. All information pertinent to the request will be retained and tracked by the data manager.

DATA ACCESS FOR RESEARCH

Author: Amit Kaura
Approvers: Jamil Mayet
Date: 09/08/2018

TROP-AF STUDY dataset

All analyses for the TROP-AF STUDY will be completed on fully de-identified data. This includes further de-identification to remove dates.

A designated clinical researcher (Amit Kaura) froze a copy of the database on 1st April 2017, so a static dataset can be used for analysis. This will be retained separately from the live database to allow reproducible analyses.

The study dataset will comprise all patients who had a troponin measured at each of the five academic centres between 2010 (2008 for University College Hospital) and 2017.

Dates will be converted to delta dates, with date zero being the date of the first troponin test. All further dates will be provided as number of days from date zero. Age will be provided in years, at the time of the first troponin test.

Date of death will be converted to the number of days since date zero. All patients will be retrospectively followed up, using routinely collected data on the NHS Spine Application, Summary Care Record, until death or censoring on 1st April 2017.

Data elements

The database model includes 156 data points, grouped into demographics, emergency department attendance and inpatient episodes, biochemistry, diagnosis, angiography, revascularization, echocardiography and mortality. Diagnostic data will be based on International Statistical Classification of Diseases and Related Health Problems (ICD) discharge codes.

DATA ANALYSIS PLAN

Author: Amit Kaura
Approvers: Jamil Mayet / Darrel Francis
Date: 09/08/2018

Study population

The study dataset will include all patients who have had a troponin measured at each of the five academic centres between 2010 (2008 for University College Hospital) and 1st April 2017.

The study population will be focussing on those with a primary diagnosis of atrial fibrillation: ICD-10 code I48: Atrial fibrillation and flutter.

We will exclude all patients with a secondary diagnosis of atrial fibrillation.

Data variables

Troponin data

In clinical practice, troponin levels are frequently dichotomised into "positive" (meaning a result above the 99th percentile of the upper limit of normal (ULN)) or "negative". Troponin levels may have a progressive relationship with prognosis, too, but the shape of this relationship is not known across the full spectrum of values and making the assumption of a linear relationship of mortality with troponin (or log troponin) may not be secure.

For these reasons, we will treat the data in two ways:

1. We will dichotomise the peak troponin level as being either positive or negative based on the ULN for each troponin assay. This makes no assumption of the shape of the relationship.
2. We will use troponin on a continuous scale by standardising the many troponin assays, by scaling the results using the ratio of the observed troponin value divided by the ULN for that particular troponin assay. For example, a patient with a troponin value of 96 using an assay which has an ULN of 40 would have a scaled result of 96/40 = 2.4 xULN.

All analyses on troponin will be performed using the peak troponin level. For patients who have a single troponin measurement, the peak troponin will be based on this measurement. In the remainder of the patients who have more than one troponin test in the same hospital episode, the peak troponin value will be defined as the highest of all measurements.

Revascularisation status

Acute revascularisation will be defined as having PCI or CABG in the time window between 48 hours before and 3 months after the first troponin measurement. This will account for patients who had revascularisation, in particular PCI, as an emergency prior to their first troponin blood test and to capture revascularisation, in particular CABG, performed as an outpatient following their index admission.

Follow-up

- Using a retrospective cohort study design, all patients will be followed up until death or censoring on 1st April 2017.
- Life status will be ascertained using routinely collected data on the NHS Spine Application, which is linked to the Office of National Statistics, and thereby to the national registry of deaths.

Outcomes

Primary outcome

The primary outcome will be all-cause mortality. The nature of the data sources means that this is the outcome that will be available and it will be available with high fidelity.

Secondary outcomes

The secondary outcomes will be:

- Angiography
- Revascularisation (coronary artery bypass grafting CABG), percutaneous coronary intervention (PCI))

Statistical Methods

Baseline data

Baseline and demographic characteristics of patients with a primary presentation of atrial fibrillation will be summarised by standard descriptive summaries:

- means (standard deviation) for continuous variables which are normally distributed
- median (interquartile range) for continuous variables which are not normally distributed

- number (percentage) for categorical variables

These characteristics will also be described for patients who did and did not undergo coronary angiography.

Comparison between angiography and no angiography groups will be made using Mann-Whitney U test or unpaired t-test for continuous variables and Chi-square test for categorical variables.

Relationship between troponin level, coronary angiography and mortality

The relationship between dichotomous troponin level (above ULN or not), or continuous troponin, and all-cause mortality will be performed using multivariate Cox proportional hazards regression modelling.

The proportional hazard assumption will be tested, with a violation indicated by a significant relationship between Schoenfield residuals of a covariate and time. If the proportional hazards assumption is violated, Cox regression analysis with time-dependent covariates will be used with follow-up time divided into time intervals within which the proportional hazard assumptions are met.

Furthermore, using Martingale residuals, if non-linearity is detected in the relationship between the log hazard and a continuous covariate, the non-linear relationship will be modelled using restricted cubic splines.

Splines will be adjusted for demographic characteristics, haematological and biochemical blood results, cardiovascular risk factors and comorbidities.

Subgroup analyses will be performed in angiography and no angiography subgroups. Kaplan-Meier survival curves will be plotted according to angiography status.

Statistical significance

All hypothesis tests will be 2-tailed. A p-value of <0.05 will be considered statistically significant. No correction will be implemented for multiple testing.

Statistical package

Statistical analyses will be performed using SPSS software version 24 (SPSS Inc., Chicago, Illinois, United States) or R 3.3.2 statistical package (the R Core Team, Vienna, Austria).

Table S1. Odds ratio of undergoing coronary angiography.

	**Odds ratio (95% CI)**	**P-value**
Male (vs female)	1.6 (1.2 – 2.1)	0.004
Diabetes mellitus	1.1 (0.7 – 1.7)	0.68
Hypercholesterolaemia	1.1 (0.8 – 1.7)	0.52
Hypertension	0.8 (0.6 – 1.2)	0.29
Aortic stenosis	2.1 (0.9 – 4.6)	0.07
Heart failure	1.1 (0.7 – 1.7)	0.67
Previous myocardial infarction	3.7 (2.6 – 5.2)	<0.0001
Malignancy	0.4 (0.2 – 0.9)	0.02
Obstructive lung disease	0.9 (0.5 – 1.6)	0.64
Positive troponin	1.5 (1.2 – 2.1)	0.003

Estimates compared to not having the disease, unless otherwise stated.

Figure S1. Odds ratio of undergoing coronary angiography.

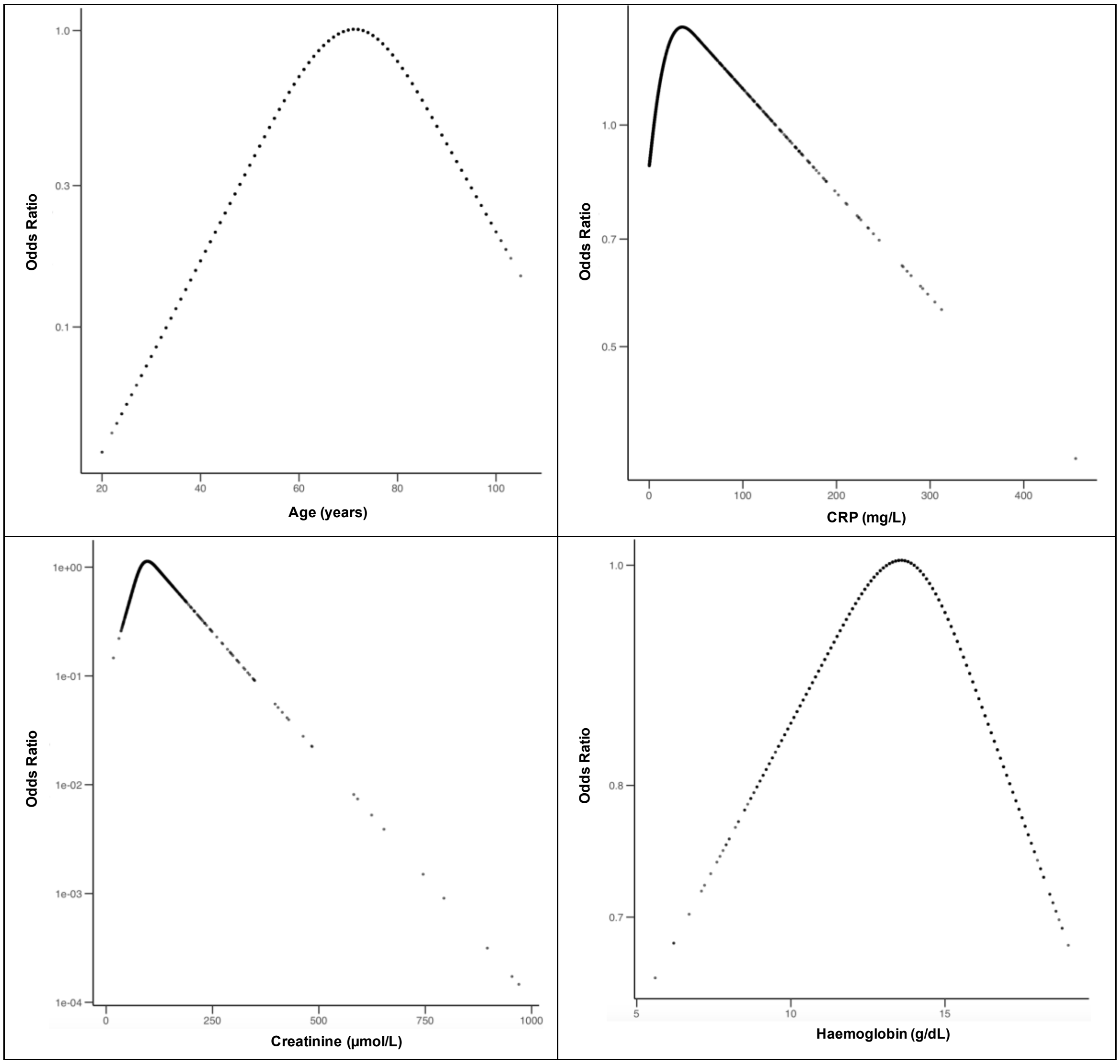

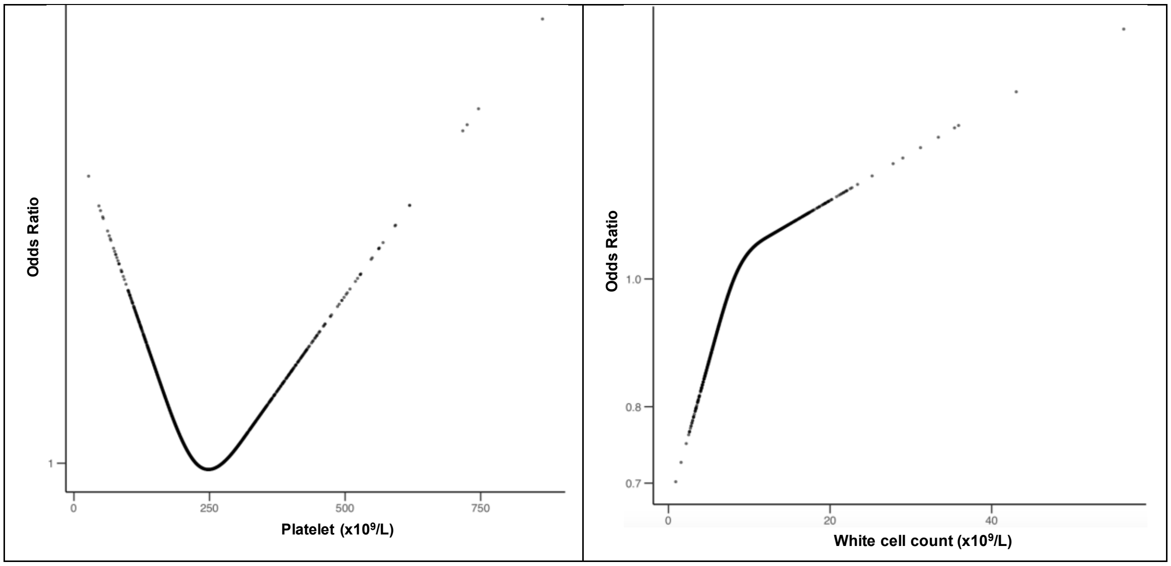
Odds Ratio
1
0
250
500
750
Platelet (x10⁹/L)
Odds Ratio
1.0
0.8
0.7
0
20
40
White cell count (x10⁹/L)

Figure S2. Odds ratio of undergoing coronary revascularisation according to troponin level.

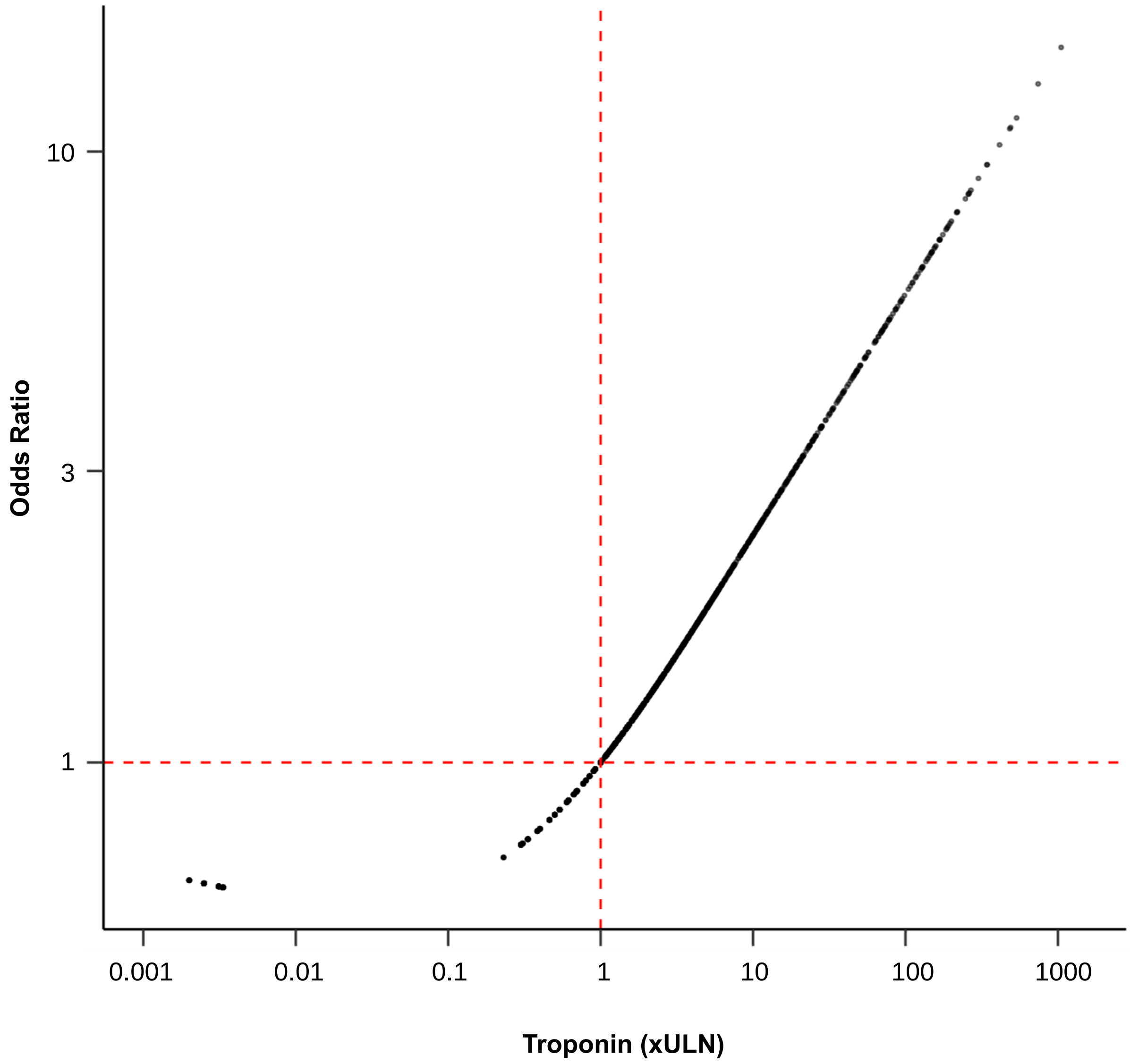

The figure shows the odds ratios of undergoing coronary revascularisation according to troponin level, where the comparator troponin level value is of 1 xULN, which is marked with the red dotted lines. ULN, 99th percentile of the upper limit of normal.

Figure S3. Multivariate* restricted cubic spline modelling of association between troponin level and hazard ratio for patients who underwent angiography without revascularisation (left) and those who did not undergo angiography (right).

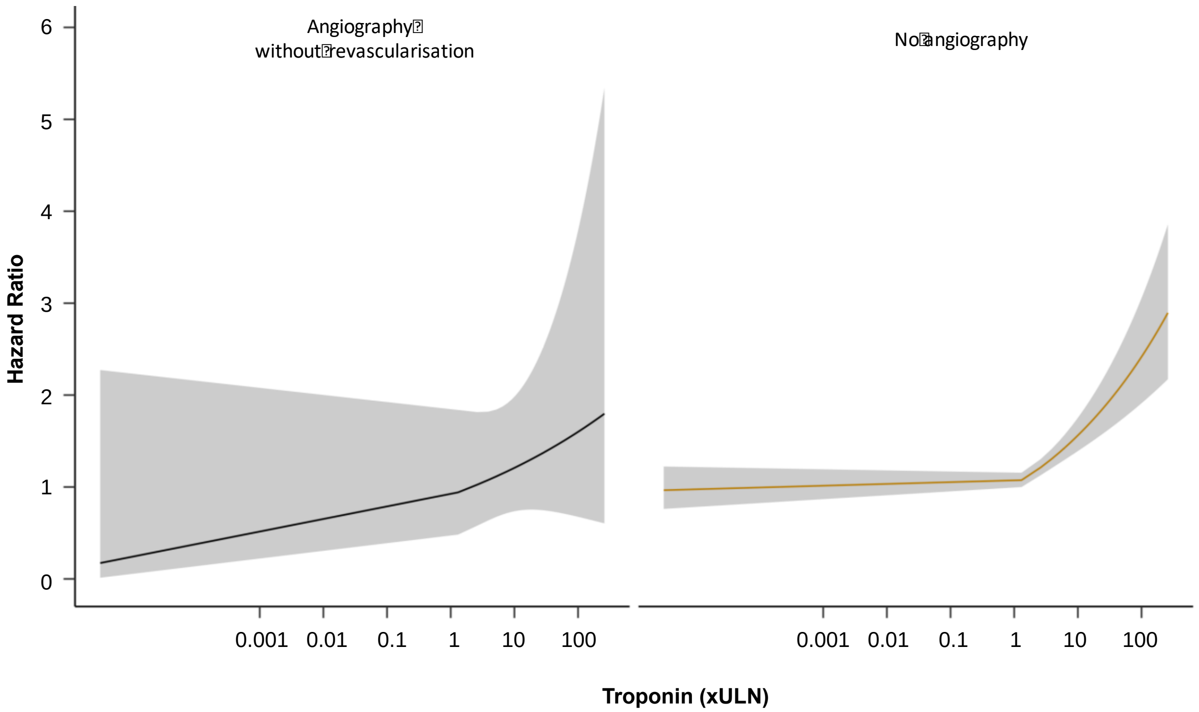

*adjustment for age, sex, C-reactive protein, creatinine, haemoglobin, platelet count, white cell count, diabetes mellitus, hypercholesterolaemia, hypertension, aortic stenosis, heart failure, previous myocardial infarction, malignancy and obstructive lung disease. The shaded area denotes the 95% confidence interval. 99th percentile of the upper limit of normal.
